# Diagnostic significance of CK19, TG, Ki67 and galectin-3 expression for papillary thyroid carcinoma in the northeastern region of China

**DOI:** 10.1186/1746-1596-6-126

**Published:** 2011-12-21

**Authors:** Qingbin Song, Deguang Wang, Yi Lou, Changsi Li, Changqing Fang, Xiangmin He, Jianhua Li

**Affiliations:** 1 Department of general surgery, The First affiliated hospital, China Medical University (Nanjing North Street), Shenyang (110001), China; 2 Department of Magnetic Resonance Imaging, No. 404 Hospital of People's Liberation Army (Baoquan Road), Weihai (264200), China; 3 Department of genetics, China Medical University (North 2. Road), Shenyang (110001), China; 4 Department of rehabilitation, The First affiliated hospital, China Medical University (Nanjing North Street), Shenyang (110001), China; 5 Department of pathology, The First affiliated hospital, China Medical University (Nanjing North Street), Shenyang (110001), China. Department of pathology, China Medical University (North 2. Road), Shenyang (110001), China; 6 Department of digestive medicine, The First affiliated hospital, China Medical University (Nanjing North Street), Shenyang (110001), China

**Keywords:** Papillary thyroid carcinoma, CK19, TG, Ki67, galectin-3

## Abstract

**Background:**

To evaluate the expression and differential diagnostic significance of CK19, TG, Ki67 and galectin-3 in papillary thyroid carcinoma (PTC) (metastatic and non metastatic), follicular adenoma and nodular goiter in patients from the northeastern part of China.

**Methods:**

441 PTC specimens and 151 other benign thyroid specimens (97 cases of nodular goiter, 54 cases of nonmalignant follicular adenoma) were collected. Immunohistochemistry for CK19, TG, Ki67 and galectin-3 was performed.

**Results:**

CK19, TG, Ki67 and galectin-3 expression was 96.37% (425/441), 82.77% (365/441), and 40.59% (179/441), 96.82% (427/441), respectively, for the PTC group and the expression of these markers in the benign thyroid lesions group was 25.83% (39/151), 79.47% (120/151), and 37.09% (56/151), 50.99% (77/151), respectively. The expression of CK19 and galectin-3 in PTC was much higher than that in the nonmalignant group (p < 0.05). However, the expression of TG, Ki67 did not differ among these two groups (p > 0.05). The diagnostic efficiency of CK19 and galectin-3 for PTC was 96.37% (537/592) and 84.63% (501/592). CK19 and galectin-3 expression rate in PTC was higher than that in benign disease cases.

**Conclusions:**

The diagnostic efficiency of CK19 for PTC was slightly better than galectin-3. The utilization of these markers combined with morphologic evaluation may be helpful in the differential diagnosis of papillary thyroid carcinoma in the northeastern region of China.

## Background

Thyroid cancer is the most common endocrine malignant tumor and encompass the most common well-differentiated papillary carcinoma (80% of all thyroid cancers) and follicular carcinoma (15%), as well as poorly differentiated carcinoma (< 1%) and anaplastic carcinoma (< 2%) [1]. PTC is the most common form of thyroid cancer, however, it is often difficult to differentiate PTC from benign papillary hyperplasia of the thyroid gland based on their morphology [2]. Some proteins' alteration was found in thyroid cancer, such as CK19, TG, Ki67, Calcitonin, TTF-1, BRAF, RET, HBME-1, SERPINA1, TfR1/CD71, FHL1 and galectin-3 [3-12].

In the recent years, a large number of molecular alterations in thyroid cancer have been used in the distinction of malignant from benign thyroid lesions. These biomarkers, such as CK19, TG, Ki67, Calcitonin, TTF-1, BRAF, RET, HBME-1, and galectin-3, have been translated into clinical practice which offered significant improvement in the preoperative diagnosis of thyroid cancer [3-6,8,11,12]. Molecular markers used in the distinction of thyroid cancer from benign thyroid lesions in the First affiliated hospital of China Medical University between 2008-2011 were showed in table 1. Among these markers, galectin-3, TG, Ki67 and cytokeratin-19 (CK-19) have been most frequently used in thyroid pathology [13-16]. In this study, we assessed four immunohistochemical markers, CK19, TG, Ki67 and galectin-3, and evaluated their diagnostic significance for papillary thyroid carcinoma in the northeastern region of China.

**Table 1:** Molecular markers used in the distinction of malignant from benign thyroid lesions

Description	Gene symbol
thyroid transcription factor-1	TTF-1
**GALECTIN 3**	**GAL3**
Calcitonin	CALCA
HEMATOPOIETIC PROGENITOR CELL ANTIGEN CD34	CD34
**keratin, type I cytoskeletal 19**	**CK19**
**Thyroglobulin**	**Tg**
**KI67 Antigen**	**MKI67**
TUMOR PROTEIN p53	P53

## Materials and methods

A total of 592 thyroid samples from the northeastern part of China collected by the Department of pathology in First affiliated hospital of China Medical University between 2008 and 2011 were used in this study. There were 441 cases in the PTC group, which included 110 men and 331 women (1:3.01), with an average age of 56 years (range 13-89 years). The diagnosis of papillary carcinomas were based on characteristic cytologic features, which includes nuclear irregularity (nuclear grooves, clearing, and increased size) and pseudoinclusions [12]. The nonmalignant group contained 151 cases (97 cases of nodular goiter, 54 cases of nonmalignant follicular adenoma) which included 38 men and 113 women (1:2.97), with an average age of 45 years (range 25-68 years). All resected specimens were fixed in 10% neutral buffered formalin (pH 7.4), embedded in paraffin, cut into 5-μm sections, and stained with hematoxylin and eosin (H&E). Informed consent was obtained from all patients that donated their specimens, and all experiments were approved by the hospital's ethics committee.

### Reagents

The antibodies included: CK19 (mouse monoclonal anti-human antibody sc-53258; 1:50; Santa cruz biotechnology, CA, USA); TG (mouse monoclonal antibody sc-53543; 1:100; Santa cruz biotechnology, CA, USA); Ki67 (mouse monoclonal antibody sc-23900; 1:100; Santa cruz biotechnology, CA, USA) and galectin-3 (mouse monoclonal anti-human anti-body clone: 9C4; 1:50; Beijing Zhong Shan Biotechnology, Beijing, China).

### Immunohistochemistry

Immunostaining has been performed by using monoclonal antibodies for CK19, TG, Ki67 and galectin-3. After deparafinisation the sections were rehydrated. Endogenous peroxidase activity was blocked by 3% hydrogen peroxide. Antigen retrieval was performed in a microwave oven for 15 min in 10 nM citrate buffer pH 6.0 for all primary antibodies. The sections were incubated at room temperature for 2 h with primary monoclonal antibodies. After washing in phosphate-buffered saline, the tissues were incubated with a biotin-conjugated secondary antibody for 1 h at room temperature. The reactions became visible after immersion of diaminobenzidine tetrahydrochloride (DAB).

### Immunohistochemical Evaluation

The cells were regarded as positive for these markers when immunoreactivity was clearly observed in their cell membrane and/or cytoplasm. For each antibody, immunoreactivity (no staining or weak staining less than 10% of the cells) was scored as negative and other immunoreactivity was scored as positive.

### Statistical Analysis

Statistical analysis was performed using SPSS V.11.5. The X^2 ^test and Fisher exact test were used for comparison of the immunohistochemistry results between the PTC and nonmalignant groups. Sensitivity (true positive/true positive + false negative), specificity (true negative/true negative + false positive), False negative rate (1-Sensitivity), False positive rate (1-specificity), and diagnostic accuracy (true positive + true negative/all positives + all negatives) of each marker were assessed in papillary thyroid carcinomas.

## Results

### Immunohistochemistry results

**Figure 1:**
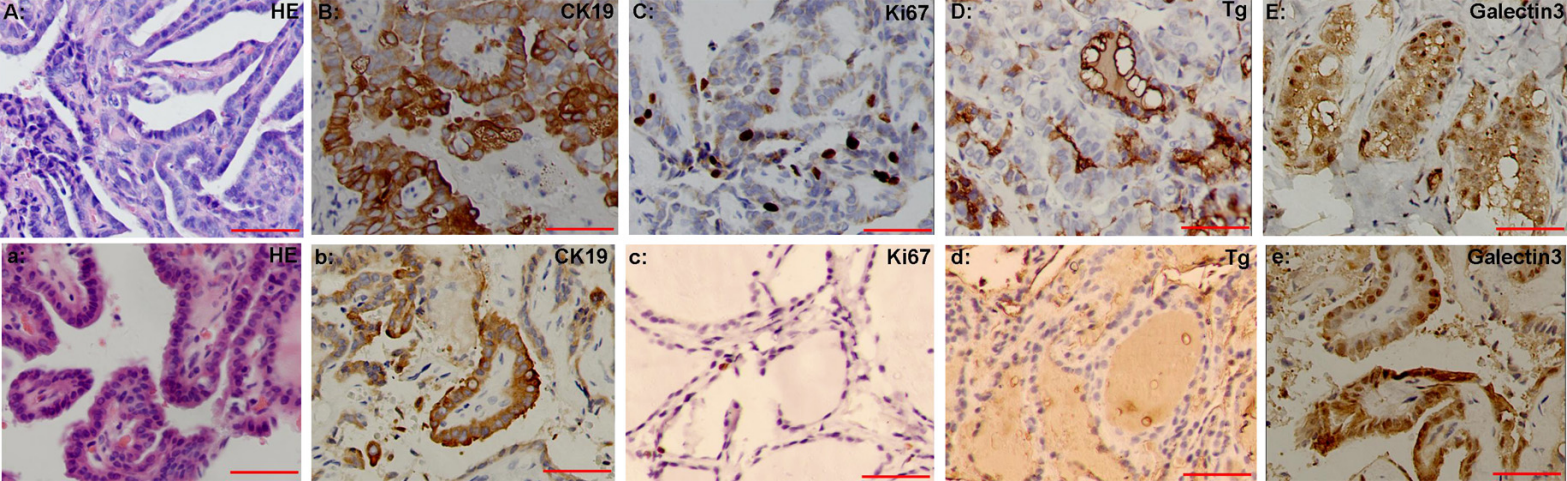
**CK19, TG, Ki67 and galectin-3 immunoreactivity in PTC and nonmalignant thyroid lesion**. A-E: HE, CK19, TG, Ki67 and galectin-3 staining in PTC; a-e: HE, CK19, TG, Ki67 and galectin-3 staining in nonmalignant thyroid lesion. Scale bar: 50 μm.

In order to evaluate the diagnostic significance of CK19, TG, Ki67 and galectin-3 expression in the distinction of PTC from benign thyroid lesions, we detected these markers expression by using immunohistochemistry. The result of immunohistochemistry was seen in Figure 1. As showed in Table 2, CK19, TG, Ki67 and galectin-3 expression was 96.37% (425/441), 82.77% (365/441), and 40.59% (179/441), 96.82% (427/441), respectively, for the PTC group and the expression of these markers in the benign thyroid lesions group was 25.83% (39/151), 79.47% (120/151), and 37.09% (56/151), 50.99% (77/151), respectively. The expression of CK19 and galectin-3 in PTC was much higher than that in the nonmalignant group (p < 0.05). However, the expression of TG, Ki67 did not differ among these two groups (p > 0.05).

**Table 2:** Immunohistochemistry results in papillary thyroid carcinoma (PTC) and other nonmalignant thyroid lesions

		Nonmalignant lesions N = 151	PTC N = 441	**X**^**2**^	P
**CK19**	**+**	39(25.83)	425(96.37)	326.17	6.56E-73
	-	112(74.17)	16(3.63)		
**Tg**	**+**	120(79.47)	365(82.77)	0.62	0.43
	-	31(20.53)	76(17.23)		
**Ki67**	**+**	56(37.09)	179(40.59)	0.44	0.51
	-	95(62.91)	262(59.41)		
**Galectin**	**+**	77(50.99)	427(96.82)	183.10	1.02 E-41
	-	74(49.01)	14(3.18)		

**Figure 2:**
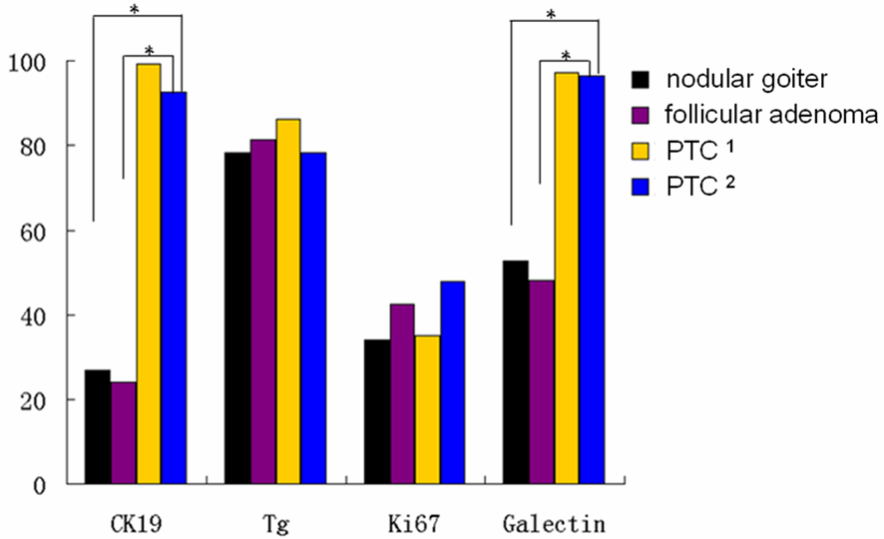
**CK19, TG, Ki67 and galectin-3 positive rate in nodular goiter, follicular adenoma, papillary thyroid carcinoma without lymphatic metastasis and papillary thyroid carcinoma with lymphatic metastasis**. PTC ^1^: papillary thyroid carcinoma (PTC) without lymphatic metastasis; PTC ^2^: papillary thyroid carcinoma (PTC) with lymphatic metastasis.

Then we analyzed CK19 and Galectin-3 expression in PTC with or without lymphatic metastasis and different benign thyroid lesions. As showed in Table 3 and Figure 2, Galectin-3 positive rate in these four groups was 52.58% (nodular goiter), 48.15% (follicular adenoma), 97.17% (papillary thyroid carcinoma without lymphatic metastasis) and 96.37% (papillary thyroid carcinoma with lymphatic metastasis), respectively. CK19 positive rate in these four groups was 26.80% (nodular goiter), 24.08% (follicular adenoma), 99.20% (papillary thyroid carcinoma without lymphatic metastasis) and 92.74% (papillary thyroid carcinoma with lymphatic metastasis), respectively. CK19 and Galectin-3 expression did not differ among nodular goiter and follicular adenoma or PTC with lymphatic metastasis and PTC without lymphatic metastasis. There was significant difference in CK19 and Galectin-3 expression between nodular goiter (or follicular adenoma) and PTC with lymphatic metastasis (or PTC without lymphatic metastasis), P < 0.05.

**Table 3:** Immunohistochemistry results of CK19 and Galectin-3 in papillary thyroid carcinoma (PTC) with or without lymphatic metastasis and other nonmalignant thyroid lesions

			nodular goiter n = 97	Follicular adenoma N = 54	**PTC**^**1**^ N = 248	**PTC**^**2**^ N = 193
**CK19**	**+**	**Female**	21(21.65)	10(18.52)	202(81.45)	121(62.69)
		**Male**	5(5.15)	3(5.56)	44(17.75)	58(30.05)
	-	**Female**	52(53.61)	30(55.56)	0(0.00)	8(4.15)
		**Male**	19(19.59)	11(20.36)	2(0.80)	6(3.11)
**Galectin**	**+**	**Female**	37(38.15)	19(35.19)	196(79.03)	122(63.21)
		**Male**	14(14.43)	7(12.96)	45(18.14)	64(33.16)
	-	**Female**	36(37.11)	21(38.89)	6(2.43)	7(3.63)
		**Male**	10(10.31)	7(12.96)	1(0.40)	0(0.00)

PTC^1^: papillary thyroid carcinoma (PTC) without lymphatic metastasis

PTC^2^: papillary thyroid carcinoma (PTC) with lymphatic metastasis

At last, diagnostics performance of CK19 in the distinction of PTC from benign thyroid lesions was compared with that of Galectin-3. As showed in Table 4, Diagnostic efficiency of CK19 (90.71%) was slightly higher than that of Galectin-3(84.63%) and the specificity and false positive rate (misdiagnosis rate) of CK19 was better than Galectin-3.

**Table 4:** Diagnostics performance of CK19 and Galectin-3 in the distinction of PTC from benign thyroid lesions

	SN	SP	FNR	FPR	DAC
**CK19**	96.37%	74.17%	3.63%	25.83%	90.71%
**Galectin-3**	96.82%	49.01%	3.18%	50.99%	84.63%

SN; Sensitivity; SP: specificity; FNR: False negative rate; FPR: False positive rate; DAC: diagnostic accuracy

## Discussion

Papillary carcinoma is the most common malignancy originating from the thyroid. Conventionally, discrimination between benign and malignant thyroid nodules is attempted by fine needle aspiration biopsy (FNAB) followed by cytological assessment [1]. Despite many advances in the diagnosis and treatment of thyroid nodules and thyroid cancer, these methods have a well known low specificity [3,4]. The searching of the reliable and repeatable immunohistochemical markers in the distinction between benign thyroid nodules and papillary thyroid carcinoma is urgent. To date, many studies have tried to classify the different biomarkers of thyroid carcinoma on the basis of their gene expression profiles. Some proteins' alteration was found in thyroid cancer, such as CK19, TG, Ki67, Calcitonin, TTF-1, BRAF, RET, HBME-1, SERPINA1, TfR1/CD71, FHL1 and galectin-3 [3-12]. Some of these proteins could be applicable to differentiate thyroid cancer from benign thyroid lesions in clinical practice. Molecular markers used in the distinction of thyroid cancer from benign thyroid lesions in the First affiliated hospital of China Medical University between 2008-2011 were showed in table 1. In this study, we assessed four immunohistochemical markers, CK19, TG, Ki67 and galectin-3, and evaluated their diagnostic significance for papillary thyroid carcinoma in the northeastern region of China.

### Ki-67

Cell proliferative activity is one of the important factors for assessing the biological behavior of carcinoma. At present, the most useful marker to evaluate cell proliferative activity is Ki-67, which is expressed in all cells except those in the G0 phase [17]. Ki-67 has potential applications in differential diagnosis between malignant and benign lesions in human neoplasms. In thyroid neoplasms, Ki-67 expression has been investigated by several groups, but it has shown conflicting. Although some of the articles support strong expression in the malignant group [18]. Another support that there is no significant difference in the Ki-67 expression between thyroid carcinoma and adenoma [19]. In this study we found Ki-67 was expressed in 37.09% benign thyroid lesions (nodular goiter or follicular adenoma) and 40.59% PTC cases and the difference of positive rate between these two groups had no statistical significance. The results suggest Ki-67 is not a suitable biomarker used in the distinction of PTC from benign thyroid lesions in the northeastern region of China.

### TG

Thyroglobulin (Tg) is exclusively produced in thyroid follicular cells or by thyroid tumors of follicular cell origin. Measurement of the serum Tg level is used to monitor patients for residual or recurrent thyroid cancer [20]. However, studies on the expression of Tg in thyroid tissues were not common and Lazar et al found TG expression was decreased in thyroid carcinomas but was normal in the other tissues [21]. In this study we found Tg was expressed in 79.47% benign thyroid lesions (nodular goiter or follicular adenoma) and 82.77% PTC cases and the difference of positive rate between these two groups had no statistical significance. The results also suggest Tg is not a suitable biomarker used in the distinction of PTC from benign thyroid lesions in the northeastern region of China.

In this study, galectin-3 and CK19 protein were analyzed by immunohistochemical method in the thyroid tissues from 592 patients with histomorphological diagnosis of nodular goiter (n = 97), follicular adenoma (n = 54), papillary thyroid carcinoma without lymphatic metastasis (n = 248), and papillary thyroid carcinoma with lymphatic metastasis (n = 93). The positive rates of Galectin-3 and CK19 in these four groups in the northeastern region of China were in complete agreement with Martins' results [22]. The difference of Galectin-3 and CK19 positive rate between PTC and benign lesions had statistical significance which suggests that the immunohistochemical study of Galectin-3 and CK19 may be potential markers for PTC.

### Galectin-3

Galectin-3 is a protein of the lectin family that has been associated with neoplastic processes in various tissues [23]. In the thyroid, expression of this protein has been described in differentiated follicular cancer, suggesting that the immunohistochemical study of galectin-3 may be a potential marker of malignancy in thyroid neoplasms [3,12,22,24-27]. Galectin-3 positive rate in these four groups was 52.58% (nodular goiter), 48.15% (follicular adenoma), 97.17% (papillary thyroid carcinoma without lymphatic metastasis) and 96.37% (papillary thyroid carcinoma with lymphatic metastasis), respectively. In a study by Bartolazzi et al., the sensitivity and specificity of galectin-3 in thyroid carcinomas were 99% and 98%, respectively [28]. In the present study, our finding about the expression of galectin-3 in thyroid carcinomas was less specificity (49.01%). This may be partially due to the subjective criteria used in assessing positive expression or genetic heterogeneity of PTC in different ethnic group.

### CK19

CK19 (Keratin 19) is a member of the keratin family. The keratins are intermediate filament proteins responsible for the structural integrity of epithelial cells. CK-19 is strongly and diffusely expressed in papillary carcinoma, whereas it is usually absent or focally expressed in benign follicular nodules [29-31]. CK19 positive rate in these four groups was 26.80% (nodular goiter), 24.08% (follicular adenoma), 99.20% (papillary thyroid carcinoma without lymphatic metastasis) and 92.74% (papillary thyroid carcinoma with lymphatic metastasis), respectively. The role of CK-19 in the diagnosis of thyroid carcinoma is controversial [32]. Schelfhout et al. have found CK-19 expression in all tumor cells of papillary carcinomas, but it was absent or only focally present in follicular carcinomas and follicular adenomas [33]. In the current study, although we found CK-19 expression in follicular adenomas or nodular goiter but the CK-19 was the most sensitive (96.37%) and specific (74.17%) marker in papillary carcinomas. Diagnostic efficiency of CK19 (90.71%) was slightly higher than that of Galectin-3(84.63%) and the specificity and false positive rate (misdiagnosis rate) CK19 was better than Galectin-3.

In conclusion, we have demonstrated four markers expressed in papillary thyroid carcinomas and found Ki-67 and Tg were not suitable biomarkers used in the distinction of PTC from benign thyroid lesions in the northeastern region of China. The utility of galectin-3 and CK-19 may provide significant contributions in the differential diagnosis of malignant thyroid tumors. The utilization of these markers combined with morphologic evaluation may be helpful in the differential diagnosis of papillary thyroid carcinoma in the northeastern region of China.

## Competing interests

The authors declare that they have no competing interests.

## Authors' contributions

QBS designed the study and wrote the protocol, YL, CSL, CQF and DGW performed research and wrote the first draft of the manuscript. XMH and JHL managed the Statistical Analysis. All authors read and approved the final manuscript.
